# Knowing Groundlessness: An Enactive Approach to a Shift From Cognition to Non-Dual Awareness

**DOI:** 10.3389/fpsyg.2021.697821

**Published:** 2021-08-06

**Authors:** Daniel Meling

**Affiliations:** ^1^Department of Psychiatry, Psychotherapy, and Psychosomatics, University of Zurich, Zurich, Switzerland; ^2^Department of Psychosomatic Medicine and Psychotherapy, Medical Center-University of Freiburg, Freiburg im Breisgau, Germany

**Keywords:** enaction, groundlessness, experience, non-duality, consciousness, cognitive science

## Abstract

The enactive approach has become an influential paradigm in cognitive science. One of its most important claims is that cognition is sense-making: to cognize is to enact a world of meaning. Thus, a world is not pregiven but enacted through sense-making. Most importantly, sense-making is not a fixed process or thing. It does not have substantial existence. Instead, it is *groundless*: it springs from a dynamic of relations, without substantial ground. Thereby, as all cognition is groundless, this groundlessness is considered the central underlying principle of cognition. This article takes that key concept of the enactive approach and argues that it is not only a theoretical statement. Rather, groundlessness is directly accessible in *lived experience*. The two guiding questions of this article concern that lived experience of groundlessness: (1) *What* is it to *know* groundlessness? (2) *How* can one *know* groundlessness? Accordingly, it elaborates (1) how this knowing of groundlessness fits into the theoretical framework of the enactive approach. Also, it describes (2) how it can be directly experienced when certain requirements are met. In an additional reflexive analysis, the context-dependency and observer-relativity of those statements themselves is highlighted. Through those steps, this article exhibits the importance of knowing groundlessness for a cognitive science discourse: this underlying groundlessness is not only the “ground” of cognition, but it also can be investigated *empirically* through lived experience. However, it requires a methodology that is radically different from classical cognitive science. This article ends with envisioning a future praxis of cognitive science which enables researchers to investigate not only theoretically but empirically the “foundationless foundation” of cognition: groundlessness.

## Introduction

Over the last few decades, the enactive approach has developed from an exotic outsider position in cognitive science to a much regarded and influential scientific paradigm (Di Paolo et al., 2010). With their first proposal for the enactive approach, Varela et al. (1991) claimed “that without the key notions of the enactive approach, cognitive science will be unable both to account for living cognition and to build truly intelligent, cognitive artifacts” (Varela et al., 1991, p. 207). And indeed, many researchers followed the direction of this bold statement. In the following decades, the key notions of the enactive approach have been applied to education, human-computer interaction, autonomous robotics, and consciousness studies (Di Paolo et al., 2010). However, the enactive approach, as outlined in *The Embodied Mind* has shown to be not static but vital. Twenty-five years after *The Embodied Mind* has been first published, Evan Thompson and Eleonor Rosch elaborated in its revised edition their correctives to the enactive approach. Those correctives have their roots in discussions that followed the first publication. This openness for correctives can be seen as “vital signs” or “indicators of the vitality of the evolutionary arc of thinking and praxis” inherent in the enactive approach (Kabat-Zinn, 2017, p. xiii). Accordingly, enactive cognitive science since then has been further developed (Thompson, 2007; Di Paolo et al., 2010). Moreover, it brought about a diversification of approaches all of which are traded under the same label “enaction,” “enactive approach,” “enactment,” or even “enactivism” (Vörös et al., 2016). Accordingly, it is helpful to clarify how the terms “enactive” and “enaction” will be used in this article.

In this article, when mentioning “the enactive approach,” I will refer to the original work by Varela et al. (1991) and to its consistent further development by Di Paolo et al. (2010) and Thompson (2007) among others. Deliberately, the work by Hutto and Myin (2013, 2017) which the authors themselves refer to as “radical enactivism” is not taken up. Also, the specific approach regarding perception called “sensorimotor enactivism” by O’Regan and Noë (2001) is not covered in this article. For a detailed elaboration of the differences between the enactive approach by Varela et al. (1991) and “radical enactivism” by Hutto and Myin (2013, 2017) see Thompson (2018).

### The Enactive Approach and Its Reply to Foundationalism: Groundlessness

What makes the enactive approach so interesting and important for the mind sciences? In the *locus classicus* of enactive cognitive science, *The Embodied Mind* from 1991, Varela, Thompson, and Rosch so importantly pointed out the *project of foundationalism* in which current cognitive science might be yet stuck in: the search for an ultimate ground, whether in the self or in the world. This is to search for something that has inherent own existence and is thereby the basis for everything else to arise. This can show up in a notion of a pregiven subject and in a notion of a pregiven world.

Being aware of such a search for ultimate grounds in cognitive science, Varela et al. (1991) proposed their alternative approach, the enactive approach. This enactive approach rejects any search for an ultimate ground. From this point of view, a search for a location of cognition is pointless. Groundlessness is what is at the heart of the enactive approach. This is what is radical and insightful about this approach to cognitive science (Vörös and Bitbol, 2017). It relinquishes any assignment of location, any search for a substantial ground that may give rise to everything else (Di Paolo, 2009). It does so to the extent to which it appreciates that everything there is springs from a dynamic of relations. As Varela et al. (1991) put it, “[e]nactive cognitive science […] require[s] that we confront the lack of ultimate foundations” (Varela et al., 1991, p. 233). This is what makes the enactive approach so unique in the landscape of paradigms in cognitive science.

### Core Themes of the Enactive Approach

Thereby, it is to bring to the foreground that such an emphasis on the idea of groundlessness is not simply out of a clear blue sky, just another concept derived from some far-off philosophical analysis. Rather, the enactive approach puts this core theme of groundlessness at the heart of its non-reductive *naturalist* framework which encompasses many other ideas that support this notion (De Jaegher and Di Paolo, 2007). This framework consists in a meshwork of ideas about life, self-organization, experience, the living body, and the world, all of which respectively exemplify the codependent arising of life, mind, body, self, and the world. None of those implies any substantial ground. Furthermore, those ideas are not radically new. Rather, the enactive approach consists in a radical *combination* of several new as well as old ideas which mutually support each other (Di Paolo et al., 2010).

One result of interlinking its central ideas on *autonomy*, *embodiment*, *emergence*, *sense-making*, and *experience* is the way the enactive approach renders cognition. Cognition in the enactive approach is understood as a deeply relational embodied action through which a cognitive system *enacts* a world of significance (Thompson, 2007, p. 13; Varela et al., 1991, pp. 205–207). Therefore, cognition is in its core to make sense of a world which is not pregiven but brought forth from within the living cognitive system’s endogenous activity. Accordingly, the enacted world is always in relation to the way the living body is organized to maintain its identity. Neither is there a pregiven world out there to be recovered by a subject, nor is there a pregiven subject projecting its own world (Varela et al., 1991, pp. 172, 173). Instead and as already mentioned, the enactive approach proposes a middle way: *groundlessness* (*śūnyatā*). Groundlessness can here be preliminarily defined as the coherent flipside of codependent arising: *whatever there is springs from a dynamic of relations, dialogically, without substantial ground*.

### Groundlessness Can Be Experienced

However, with regard to groundlessness, Varela et al. (1991) point out an important aspect to consider:

*All of our activities depend on a background that can never be pinned down with any sense of ultimate solidity and finality. Groundlessness, then, is to be found not in some far off, philosophically abstruse analysis but in everyday experience (Varela et al., 1991, p. 144)*.

This points toward a crucial point of departure of this article. Groundlessness is not just another philosophical concept but rather something *experiential*. It can be directly experienced. More than that, it is not only possible but even crucial to experience groundlessness and to learn to live with groundlessness as Varela et al. (1991) point out: “our historical situation requires not only that we give up philosophical foundationalism but that we learn to live in a world without foundations” (Varela et al., 1991, p. 218).

Unless groundlessness is lived and experienced, there remains a strong tension between the *belief* in groundlessness (or philosophical position thereof) and what one *does*, i.e., grasping after a ground in the concept of groundlessness. This tension is elucidated in recognizing that the insight into an absence of an absolute ground alone does *not* necessarily change the deeply rooted habitual tendencies of the mind to grasp after grounds. This grasping mind can therefore also cling to the *absence* of an absolute ground. Thereby, it can regard everything else as just an illusion (Varela et al., 1991, p. 143).

This scenario of a mind grasping after groundlessness as just another ground can be prevented and overcome. One does so by learning to *live* in a world without foundations rather than just *knowing about* this lack of foundations. Rather than clinging to that knowledge *about* groundlessness, one lets go of clinging to *any* kind of knowledge. One learns to live with uncertainty.

This difference between knowing groundlessness as a philosophical idea and knowing groundlessness as a lived experience is beautifully put in Thompson’s (2004) statement on Varela’s view concerning the difference between scientific theory and direct experience:

*He never lost sight of this point that the mind–body problem is not only a philosophical problem, or a scientific problem, but also a problem of direct experience. The problem could be put this way. It’s one thing to have a scientific representation of the mind as “enactive” – as embodied, emergent, dynamic, and relational; as not homuncular and skull-bound; and thus in a certain sense insubstantial. But it’s another thing to have a corresponding direct experience of this nature of the mind in one’s own first-person case (Thompson, 2004, p. 382)*.

Accordingly, practicing to directly experience the world as groundless is not only possible but of utmost importance. This importance is even more the case for people who explicitly operate with the *idea* of groundlessness and who are thereby at risk of clinging to this idea of groundlessness. To great extent, this is a risk in operating with the enactive approach.

Therefore, a *form of practice* is required in which one becomes familiar with experiencing the groundless nature of all phenomena, including enactive ideas and the concept of groundlessness itself. In this regard, Varela et al. (1991) highlighted *Buddhist* mindfulness/awareness meditation as one potential means. The aim of this practice is to develop a direct and stable insight into forms of grasping and to let go of such tendencies to grasp (Varela et al., 1991, p. 144). Such a relation between a practice and a lived experience of groundlessness is pointed out once more by Varela et al. (1991): “it is said that emptiness is a natural discovery that one would make by oneself with sufficient mindfulness/awareness—natural but shocking” (Varela et al., 1991, p. 225).

What is referred to as emptiness in this case can translate into groundlessness or *śūnyatā*. This groundlessness as a natural discovery is to be seen as equivalent to the *lived experience of groundlessness* in contrast to a merely philosophical and abstract notion of it. Moreover, Varela et al. (1991) related this discovery of groundlessness to a specific element of mindfulness/awareness practices, namely non-grasping: “[b]y progressively learning to let go of these tendencies to grasp, one can begin to appreciate that all phenomena are free of any absolute ground and that such “groundlessness” (śūnyatā) is the very fabric of dependent coorigination” (Varela et al., 1991, p. 144).

Not only is groundlessness the fabric of such codependent arising but it is also to be understood as a certain mode of knowing. This is well expressed by Varela et al. (1991): “[k]nowing śūnyatā (more accurately knowing the world as śūnyatā) is surely not an intentional act. Rather (to use traditional imagery), it is like a reflection in a mirror—pure, brilliant, but with no additional reality apart from itself” (Varela et al., 1991, p. 225). This exact point is beautifully stressed in Rosch’s (2017) introduction to the revised edition of *The Embodied Mind*. She argues for groundlessness being a mode of enaction different from a usual sense-making. Eleonor Rosch distinguishes them in *phase 1 enaction* (sense-making) and *phase 2 enaction* (reflexive awareness).

This distinction between phase 1 enaction and phase 2 enaction is crucial to this article. In *phase 1 enaction*, the knowing of a world is related to performing actions relevant to the self-maintenance of the living body. This living body, in turn, is understood as a self-organizing system which co-dependently emerges. Thereby, its aspect of “adaptivity” makes phase 1 enaction normative. Moreover, phase 1 enaction is dualistic: it involves a distinction between subject and object, between observer and observed. It is purposive and related to getting the good, shunning the bad and ignoring the indifferent from the perspective of the subject pole of the dualism. While phase 1 enaction is accordingly normative, these norms themselves are brought forth in the process of the living being’s embodiment. Accordingly, norms are not inherently existent. *Phase 2 enaction*, on the other hand, is an alternative mode of knowing that is *neither* based on an observer and an observed *nor* on an embodied enactment of norms. In this phase 2 enaction the mind is “neither absorbed nor separated but simply present and available” (Rosch, 2017, p. xl). Furthermore, this experience is simple and self-known without any sense of an observer: “this is the mind that can actually know firsthand the groundlessness of the enacted edifice in which humans live […], thereby clearing the way for transformative wisdom to emerge” (Rosch, 2017, p. xl). Therefore, as phase 2 enaction points out a non-intentional, simple, content-free mode of experiencing, it appears challenging to give a positive definition of it. Rather, a more appropriate path for communicating it might be found in guiding toward the direct lived experience of phase 2 enaction. This is why this article moves toward a pragmatic orientation of pointing out a path that conceptualizes from an enactive standpoint what is required for a living system to directly experience phase 2 enaction or even groundlessness. Moreover, this conception of phase 2 enaction is in close resemblance with the notion of sustained, non-propositional meta-awareness as introduced by Dunne et al. (2019). Therefore, phase 2 enaction is a non-dual mode of knowing that allows for a direct experience of groundlessness.

### Research Questions and Aim

The most important ideas from this introduction can be summarized in three steps. First, groundlessness, as we have seen, is theoretically central for the enactive approach and for understanding cognition at large. Second, groundlessness can and also needs to be lived in one’s own experience. One way to enable such experience is through certain forms of meditation that include a letting-go of one’s tendency to grasp. Third, groundlessness appears to be a certain mode of knowing which is different from everyday usual sense-making: it is non-intentional, lacks any subject-object distinction, and is therefore non-dual.

Regarding this, two crucial questions are still open:

(1) What is knowing groundlessness and how is it different from sense-making?
(2) What does a shift from adaptive sense-making (phase 1 enaction; dual knowing) to knowing groundlessness (recognizing phase 2 enaction; recognizing non-dual knowing) require?

These two research questions will guide us through this explorative theoretical article. However, why do I address these two questions from the point of view of the enactive approach?

Compared to the beginnings of the enactive approach, those two questions can be approached today with a more elaborate set of conceptual tools. Since *The Embodied Mind* has been published in 1991, the enactive approach has been developed further in many ways as a vital active field of theory and research (Di Paolo et al., 2010; Rosch, 2017, p. xlviii). Accordingly, the conceptual repertoire of the enactive approach has been expanded and fine-tuned (cf. Di Paolo, 2005; Thompson, 2007; Di Paolo et al., 2010). However, to my current knowledge this rich conceptual repertoire of the contemporary stage of the enactive approach has not yet been applied to a description of this *mode of “knowing the world as śūnyatā*.*”* At the same time, this *knowing of groundlessness* is one of the deepest sources of the enactive approach. Therefore, such a description of a shift to knowing groundlessness which includes contemporary enactive concepts such as sense-making, autonomy, adaptivity, and dynamic co-emergence has the rich potential to contribute to the enactive approach as a theory.

The goal of this article is therefore to describe the process in which a mode of adaptive sense-making (phase 1 enaction; dual knowing) transforms into a mode of *knowing groundlessness* (recognizing phase 2 enaction; recognizing non-dual knowing). This process is to be described in terms of the conceptual repertoire provided by contemporary enactive cognitive science.

This description may be valuable in two ways. First, it is a re-emphasis of groundlessness being at the heart of the enactive approach. Second and more importantly, it highlights the lived epistemological aspect of groundlessness. Groundlessness is not only an idea but an experience. Accordingly, this article provides a principled account of groundlessness that is integrated as an *experience* into the framework of the enactive approach. This integration of knowing groundlessness into the enactive framework opens the discourse for an alternative mode of knowing that is beyond the usual enactive conceptualization of cognition as sense-making.

From there, the requirements of a momentary transition from sense-making to knowing groundlessness are described. This description adopts ideas from the rich contemporary repertoire of the enactive approach.

Why is this important? The purpose of exploring knowing groundlessness and the transition from sense-making to knowing groundlessness is two-fold. First, groundlessness is at the *core* of cognition. To open the discourse for knowing groundlessness has potential for illuminating the deeper dependency and relativity of cognition. It enables crucial insight and demands from scientific practice to become reflexively aware of itself. Second, knowing groundlessness is related to well-being. As “grasping after a ground […] is the deep source of frustration and anxiety,” to release that grasp means to alleviate frustration and anxiety at its source (Varela et al., 1991, p. 143). This makes the investigation of knowing groundlessness a deeply ethical endeavor.

The aim of this article is to *theoretically* frame knowing groundlessness and the transformative process leading to it. By this, I most humbly hope to contribute to opening up theoretical cognitive science to at least *conceiving* a non-dual kind of knowing via the enactive approach.

## The Transition From Sense-Making to Knowing Groundlessness

The main goal of this section is to describe the transition from adaptive sense-making (phase 1 enaction; dual knowing) to knowing groundlessness (recognizing phase 2 enaction; recognizing non-dual knowing). In this we encounter three elements: (1) adaptive sense-making as the point of departure, (2) knowing groundlessness as the point of arrival, and (3) the transition process between the two.

A description of knowing groundlessness in relation to the whole enactive terminology (i.e., sense-making, adaptivity etc.) is to be contributed by tackling the first research question (RQ1). Also, the transition from adaptive sense-making to knowing groundlessness is yet to be explored. Preliminarily, we have seen that it requires to let go of grasping. A description of the transition from adaptive sense-making to knowing groundlessness in relation to the enactive framework is to be contributed by tackling the second research question (RQ2). Therefore, I am going to elaborate knowing groundlessness and the transition from adaptive sense-making to knowing groundlessness in enactive terms.

In order to approach those two research questions, a point of departure and a rough direction are required. The point of departure is adaptive sense-making. The rough direction is a decrease of adaptive sense-making. The following quote, which I already referred to earlier, provides a corresponding hint why a decrease of sense-making might be required for a transition to knowing groundlessness: “[k]nowing sunyata (more accurately knowing the world as sunyata) is surely not an intentional act. Rather (to use traditional imagery), it is like a reflection in a mirror—pure, brilliant, but with no additional reality apart from itself” (Varela et al., 1991, p. 225).

This quote provides the crucial hint: What is present in sense-making is not present in knowing groundlessness. While sense-making is intentional, knowing groundlessness is not. While sense-making includes a perspective and a respective subject-object structure, knowing groundlessness does not. While sense-making is affect-driven with respect to adaptivity, knowing groundlessness is not. Thereby the most important features of sense-making are absent in knowing groundlessness. Therefore, a decrease of sense-making becomes the guiding direction: groundlessness can be known when sense-making has ceased.

This section tackles the two research questions on two distinct levels of analysis. (A) The *first level of analysis* is structured into five parts. First, the enactive notion of sense-making is presented as the point of departure. Second, the first stage in approaching groundlessness is presented. Sense-making can be decreased via decreasing adaptivity. This comes with an important issue: adaptivity and sense-making must be known in order to let them go. This leads to the next stage. Third, the second stage in approaching groundlessness is presented. For their decrease, adaptivity and sense-making need to be known via phase 2 enaction. It is an alternative mode of knowing. It is non-dual, reflexive, and self-known. This non-adaptive reflexive awareness of the current adaptivity and sense-making enables them to run down. In a gap of sense-making, non-dual reflexive knowing (phase 2 enaction) can recognize itself. Fourth, the point of arrival is presented. Groundlessness is known when phase 2 enaction knows itself, non-dually. Fifth and finally, the conclusion of the first level of analysis is presented. The answers to the two research questions are summarized. (B) The *second level of analysis* comprises a reflexive turn. The provided description is itself based on a lived state of adaptive sense-making. To describe the transition from sense-making to knowing groundlessness as provided in the first level of analysis is to describe it from a point of view that embodies adaptive sense-making. In the second level of analysis, I emphasize that an alternative view from the perspective of knowing groundlessness would contradict the description of the first level of analysis. Finally, the insights from this section are summarized.

### First Level of Analysis

#### Point of Departure: Sense-Making

The transition to knowing groundlessness starts from adaptive sense-making. As this notion of adaptive sense-making is crucial for the subsequent steps, let us summarize the key points from the enactive approach: *sense-making* is the process by which an organism, based on the characteristics of its individuation activity (autonomy), makes meaning and constitutes a world of significance for itself. Cognition is exactly this creation of meaning.

Through its *autonomy*, a living system enacts significance on its world and transforms it accordingly into an environment of meaning and valence. It regulates its couplings with its environment as it aims at the continuity of its self-generated identity. This initiates the regulation (De Jaegher and Di Paolo, 2007, p. 488; Varela, 1997). Therefore, an organism establishes a perspective on the world with its own normativity. This means that some interactions are experienced as good, others as bad, and the rest as irrelevant to its continuous self-generation. Accordingly, sense-making is always a relational and even *affect-laden* process based on biological autonomous organization (De Jaegher and Di Paolo, 2007, p. 488). In this way, autonomy is deeply related to sense-making as it gives rise to a *reference point* or *perspective* to which interactions with the environment gain significance. On this basis, all sense-making (phase 1 enaction) is the enaction of norms with respect to the system’s autonomy. Also, it is the corresponding regulation with respect to those norms. Hence, sense-making always requires autonomy and adaptivity.

*Adaptivity*, as introduced by Di Paolo (2005), refers to the “capacity of an organism to regulate itself with respect to the boundaries of its own viability” (Di Paolo, 2005, p. 430). Hence, an organism can avoid situations that endanger its autonomy and can seek those that support its maintenance. While autonomy provides an identity as the center of a perspective, adaptivity allows the organism to regulate its encounters in a graded manner with respect to what is better or worse from this provided perspective.

Three implicit assumptions need to be emphasized at this point. First, sense-making is based on adaptivity. This is to enact norms and regulate one’s interactions with the environment for sustaining one’s autonomous identity. This is a crucial point because it reveals that sense-making is the organism’s *answer* to perturbations and potential threats. Accordingly, sense-making is what the organism applies as part of a *solution* in the face of a perturbation. In our case in which we are going to look for a *decrease* in sense-making, exactly this solution itself has become dysfunctional: if an organism makes sense of a perturbation, it adds sense-making. However, when the specific taskset involves a decrease in sense-making, this exact reaction of the organism turns out to be adverse to approaching this specific goal. In other words, the solution has become a problem. The application of the same solution would consequently lead to a stronger manifestation of the same problem. Accordingly, we will see later how to go about such a decrease of sense-making without adding more of it. Second, sense-making does not only occur at one single level but on several different levels simultaneously. If one sense-making pattern ceases, another pattern may become salient and dominant. Third, sense-making is not static but dynamic. It involves a dynamic evolvement of transient and precarious patterns which are only semi-stable for a certain duration.

On the basis of this adaptive sense-making as the point of departure, we will now tackle the afore-mentioned research questions and synthesize an enactive framework of the transition from adaptive sense-making to knowing groundlessness.

#### Stage 1: Less Sense-Making

In this first stage, we are guided by the following question: how can an autonomous adaptive system decrease sense-making?

As we have seen, sense-making requires both, autonomy and adaptivity. If there is no autonomy, there cannot be sense-making. Likewise, if there is no *adaptivity*, there cannot be sense-making as well. As autonomy constitutes the condition for the organism to be alive, a decrease of sense-making via decreasing autonomy would involve the death of the organism: a dead organism cannot know, obviously. However, the aim is to decrease sense-making while keeping the system alive. Therefore, the better option is to decrease sense-making via decreasing adaptivity.

What happens when a system decreases its adaptivity? This means it appraises less. It lets go of approach/avoidance tendencies. We have good reason to assume that the respective enacted meaning would also decrease as it is based in this particular appraisal and regulation activity.

**FIGURE 1:**
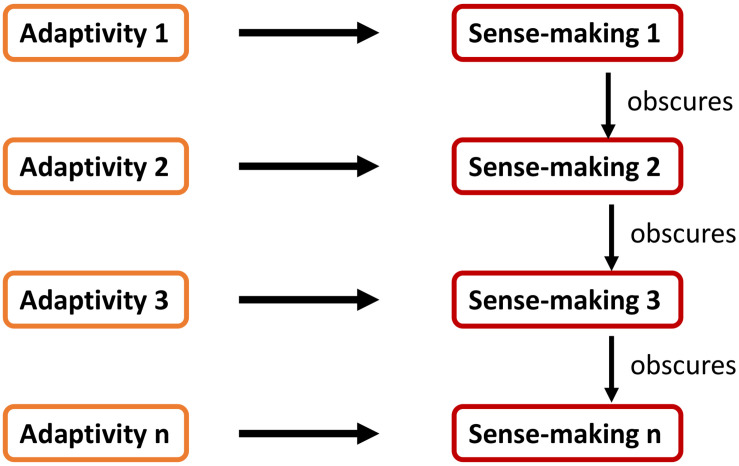
A simplified model of various sense-making processes that depend on respective acts of adaptivity; in this case, sense-making1 is most apparent in the agent’s experience.

To illustrate this, let us evoke a scenario with various sense-making processes. Remember that sense-making occurs on several different levels simultaneously: if one sense-making pattern ceases, another pattern may become salient and dominant (cf. section “Point of Departure: Sense-making”). For illustration purposes, the distinctness of those sense-making patterns will be highlighted by marking each of those with a respective index. Accordingly, we can distinguish sense-making_1_, sense-making_2_, and sense-making_3_ as different patterns based on their respectively different adaptivity acts, namely adaptivity_1_, adaptivity_2_, and adaptivity_3_ (Figure 1).

**FIGURE 2:**
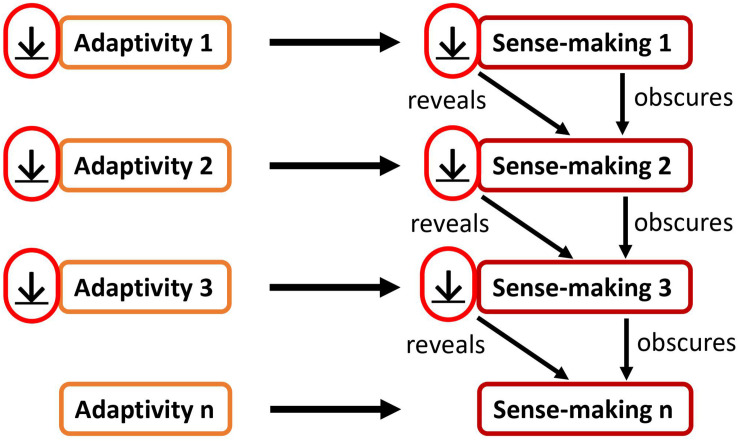
The decrease of an adaptivity act decreases the respective sense-making process to which it gives rise; as a result, a subtler sense-making act becomes apparent.

If adaptivity_1_ is decreased, also its according sense-making_1_ is decreased (Figure 2). As a result, a more subtle sense-making_2_ and its according adaptivity_2_ can become apparent. If, in turn, this adaptivity_2_ is then decreased, also sense-making_2_ is decreased. Again, as a result, a more subtle sense-making_3_ and its according adaptivity_3_ can become apparent. This process can go on for quite some time while the living system encounters increasingly subtle sense-making activities and their according adaptivity. This process may continue until there is a gap of sense-making and adaptivity. In such a gap, the living system could then experience a moment of groundlessness unobscured by sense-making.

**FIGURE 3:**
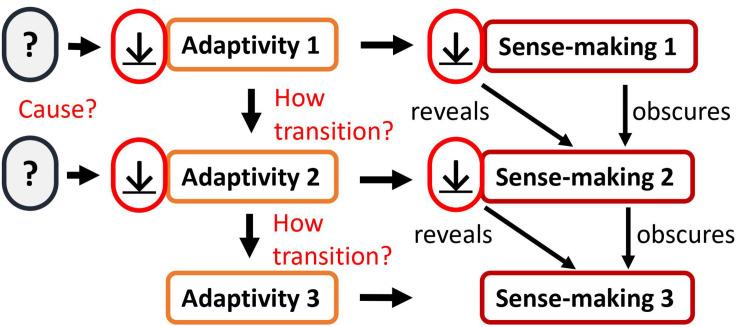
The main gaps of the “stage 1” approach as it is outlined in Figure 2: (1) What causes the respective adaptivity act to decrease? (2) How does the cognitive system transition from decreasing one act of adaptivity to decreasing another subtler act of adaptivity?

However, there is a *problem* with this approach. While the overall approach makes sense, something is lacking: how can adaptivity and sense-making be decreased when they themselves are *unknown*? As we *are* in sense-making and in adaptivity, we are blind for them. We are blind for the current sense-making from which we are looking out. To know the sense-making itself is usually irrelevant for our getting-and-avoiding aims (adaptivity). Therefore, it is logically consistent that sense-making is usually ignored by sense-making itself. However, we have seen that two steps are necessary in order to decrease sense-making. First, we would need to decrease adaptivity. Second, we would need to transition from decreasing adaptivity_1_ to decreasing adaptivity_2_ to decreasing adaptivity_3_ and so on. This is required for encountering more and more subtle sense-making activities and for dissolving them (as illustrated in Figure 2). At some point this may lead to a gap of sense-making. However, this comes with a problem which is highlighted in Figure 3: first, if we do not know the current sense-making and adaptivity, how could it then be decreased? Second, if we do not know the current sense-making and adaptivity, how could we then transition from decreasing adaptivity_1_ to decreasing adaptivity_2_ to decreasing adaptivity_3_ and so on? Therefore, we need to expand this model: a living organism needs to *know* and *directly discover* its own current sense-making activity (not content) and the according adaptivity in order to decrease them.

#### Stage 2: Know Thyself

In stage 1, we came across the necessity to know the current knowing itself. This turned out to be required for reducing sense-making. But how can an autonomous adaptive system know its own adaptivity and sense-making?

**FIGURE 4:**
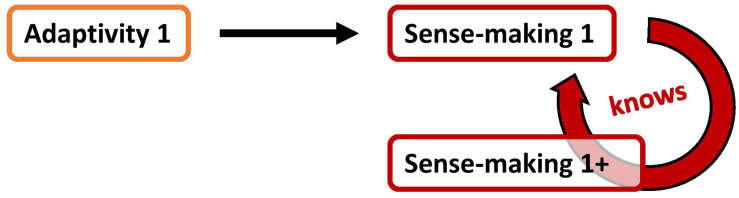
The “sense-making-of-sense-making” approach; the current sense-making act is recognized via an additional act of sense-making.

In our current picture of the enactive approach, all cognition and respectively all knowing is sense-making (phase 1 enaction). Therefore, a first consequent intuition would be to propose a sense-making *of* sense-making itself. Accordingly, the current sense-making (and potentially also its underlying adaptivity) is thereby recognized by an additional act of sense-making_1__+_. This is illustrated in Figure 4.

**FIGURE 5:**
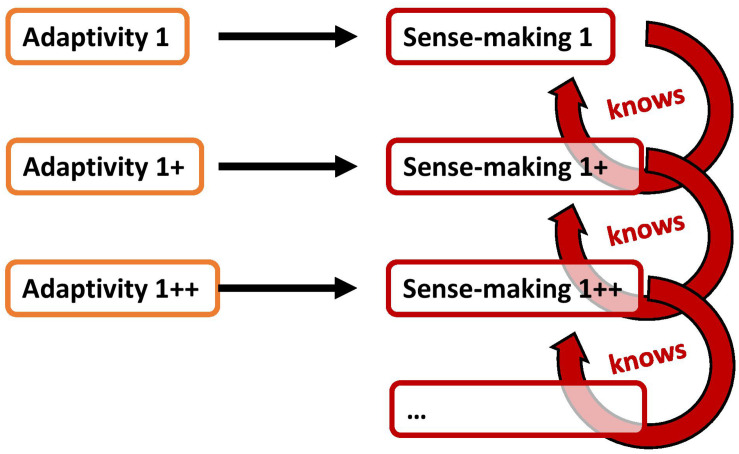
The “sense-making-of-sense-making” approach leads to an increase of sense-making and adaptivity acts.

However, also in this approach we encounter an important *problem*: the subsequent act of sense-making also requires autonomy and adaptivity. Therefore, it brings with it a next act of adaptivity_1__+_. Those additional sense-making_1__+_ and additional adaptivity_1__+_ need also to be known. Therefore, it needs another act of sense-making, a sense-making_2__+_, and adaptivity_2__+_. Apparently, we gain more and more sense-making and adaptivity (Figure 5). In principle, this approach of “sense-making of sense-making” leads to an *infinite regress*.

The result of this “sense-making-of-sense-making” approach is directly opposite to our aim of *decreasing* sense-making through decreasing adaptivity. So, how does an autonomous adaptive system know its current adaptivity and sense-making if “sense-making of sense-making” does not work? The reply is simple: it requires a mode of knowing that is not sense-making: phase 2 enaction.

This non-dual reflexive knowing (phase 2 enaction) is an alternative mode of knowing (Rosch, 2017, p. xxxix). Therefore, it is well characterized in its opposition to adaptive sense-making (phase 1 enaction). As we have already seen, usual sense-making is based on adaptivity. Its foundation is the normativity that arises from the system’s aim to preserve its maintenance. Sense-making is based on tendencies of approach and avoidance. It is dualistic in the sense that it involves an experience of a subject-object separation. While being guided by the approach-avoidance tendencies inherent in adaptivity, the organism’s experience is absorbed in the object. Its attention is biased. It discerns between the good, the bad, and the irrelevant. The organism pays attention to the good and to the bad and ignores the irrelevant. However, these irrelevant aspects of the environment are, principally, aspects of the organism’s perceivable environment which it simply does not perceive more explicitly as they may not have any relevance to the preservation of its autonomy.

In contrast, non-dual reflexive knowing (phase 2 enaction) lacks such discernment. Although sense-making and adaptivity might be present from a previous act of such phase 1 enaction, this “knowing groundlessness” would be initiated through an *experiencing* that just remains open for everything without any preferences. Such non-dual experiencing does not discern between the good or the bad. It does not ignore the irrelevant. It is simple and open. Therefore, it must be effortless. It lacks any agenda or preferences. Even the preference of a non-dual experiencing over a dual sense-making would simply add an adaptive act. This would lead to another sense-making act that obscures the already present non-dual reflexive knowing. While it remains that open, it can also illuminate what is already there itself: if there is adaptivity and sense-making, it can experience them without being obscured by them.

As the non-dual reflexive knowing (phase 2 enaction) is undiscerning and open for everything, it is non-adaptive. Therefore, with non-dual reflexive knowing (phase 2 enaction) alone the *precarious* activity of adaptivity and sense-making cannot sustain. Accordingly, current acts of adaptivity and their respective sense-making tend to run down. This includes various kinds of sense-making activities, including the recall of memories and the enactment of a notion of one’s personhood. Consequently, as these sense-making and adaptivity activities decrease, the experience becomes increasingly memoryless, impersonal, and overall content-free. What is left is non-dual reflexive experiencing (phase 2 enaction).

Thereby, in a transition from adaptive sense-making to knowing groundlessness each moment’s sense-making is recognized via non-dual reflexive knowing (phase 2 enaction). The result is that adaptivity and sense-making cease. In this openness, the reflexive non-dual knowing (phase 2 enaction) can recognize itself.^1^ This is knowing groundlessness.

#### Point of Arrival: Knowing Groundlessness

Knowing groundlessness is phase 2 enaction knowing itself, unobscured by adaptive sense-making. Thereby, on the path toward knowing groundlessness phase 2 enaction turned out to be not only the *method* but in some way also the *destination*. This framing of knowing groundlessness is beautifully echoed by Eleonor Rosch:

*[w]hat Buddhist practices have to contribute to this conundrum is that there is a different mode of knowing altogether in which the mind is neither absorbed nor separated but simply present and available. There is no longer that observer claimed in the first chapter here; experience is simple and self-known. This is the mind that can actually know firsthand the groundlessness of the enacted edifice in which humans live (chapter 10), thereby clearing the way for transformative wisdom to emerge (hinted at in chapter 11) (Rosch, 2017, pp. xl–xli, emphasis added).*

This passage clearly reflects the difference between non-dual reflexive knowing (phase 2 enaction) and knowing groundlessness (*recognizing* phase 2 enaction). Non-dual knowing is the kind of knowing that does not involve any sense-making or adaptivity. It is reflexive awareness. It might be already present in a moment of sense-making but unrecognized. In contrast, “knowing groundlessness” describes the experience in which this non-dual reflexive awareness is *recognized*. In this knowing groundlessness the reflexive awareness is unobscured by any act of sense-making. It finally can recognize itself, non-dually. This unobscured self-recognition of reflexive non-dual experiencing is what I currently understand as the point of arrival we were heading to: a moment of knowing groundlessness.

#### Conclusion: The Research Questions Revisited

In the first level of analysis we explored a description of the transition process from adaptive sense-making to knowing groundlessness. Now we can summarize it with respect to the research questions.

The first research question RQ1 asked about the direct experience of groundlessness: what is knowing groundlessness and how is it different from sense-making?

In the first level of analysis we arrived at a definition of knowing groundlessness: knowing groundlessness is phase 2 enaction knowing itself, unobscured by phase 1 enaction (adaptive sense-making). Thereby, knowing groundlessness is based on phase 2 enaction, not on phase 1 enaction. Phase 2 enaction is an alternative mode of knowing. While phase 1 enaction is intentional, phase 2 enaction is not. While phase 1 enaction includes a perspective and a respective subject-object structure, phase 2 enaction does not. While phase 1 enaction is affect-driven with respect to adaptivity, phase 2 enaction is not. Thereby, phase 2 enaction is non-intentional, self-known, and non-discerning. In a gap of sense-making, this phase 2 enaction is unobscured. In such a moment it can recognize itself. This unobscured self-recognition of phase 2 enaction is knowing groundlessness.

The second research question RQ2 asked about the process of a lived transition from sense-making to knowing groundlessness: what does a shift from adaptive sense-making (phase 1 enaction; dual knowing) to knowing groundlessness (recognizing phase 2 enaction; recognizing non-dual knowing) require?

In the first level of analysis we arrived at a description of this transition process. It exhibits two requirements: non-adaptivity and non-dual reflexive knowing (phase 2 enaction). Non-adaptivity is the first requirement. It means that a living organism refrains from regulating itself with respect to the boundaries of its own viability. It does not appraise its experiences as good, bad, or irrelevant. Accordingly, it does not approach, avoid, or ignore anything. Closer to experiential terms, this can be understood as unconditional acceptance. Non-dual reflexive knowing (phase 2 enaction) is the second requirement. It means that a living organism taps into its capacity for phase 2 enaction. Via this phase 2 enaction, it recognizes its current acts of sense-making and adaptivity. Closer to experiential terms, this can be understood as effortlessly becoming aware of one’s thought and affect. One recognizes one’s current thought *as* a thinking act. Likewise, one recognizes one’s current affect *as* an affective act. Importantly, this recognition lacks a sense of observer and observed. It is non-dual.

Those two requirements are met in the transition process from adaptive sense-making to knowing groundlessness. In this transition process the current acts of adaptivity and sense-making are experienced. Simultaneously, there is mere acceptance through which no adaptivity is added. Therefore, sense-making is decreased. Accordingly, when this is continued, acts of adaptivity and sense-making progressively decrease. Thereby, there is progressively less obscuration of the already present non-dual reflexive experiencing (phase 2 enaction). This may lead to a gap of sense-making in which phase 2 enaction can recognize itself. Experience experiences itself, non-dually. This is a moment of knowing groundlessness.

### Second Level of Analysis

In the first level of analysis we arrived at a description of knowing groundlessness and of the transition process leading to it. In the second level of analysis we will now turn our attention reflexively to this description itself: it is enacted. First, the provided description is context-dependent and observer-relative. Second, it comes from *within* acts of adaptive sense-making (phase 1 enaction). Third, this description is inappropriate from *within* the “perspective” of knowing groundlessness. Fourth, none of the two perspectives is ultimately true. They are useful in different contexts.

#### The Enacted Description Is Context-Dependent and Observer-Relative

In the first section we have seen that cognition is sense-making. To cognize is to enact a world. This world-making is based on the cognizing system’s autonomous organization and its history of structural couplings. As this applies to the encountered world it also applies to any statement about the world. Thereby, any statement about the world is observer-relative and context-dependent.^2^ No description whatsoever can be true independent of its observer and its context. This itself is a statement. Accordingly, it cannot be ultimately true either.

This observer-relativity and context-dependency of any statement can be illustrated by revealing each statement’s incoherence. This incoherence of statements can be rationally shown by means of the so-called *neither-one-nor-many* argument. In certain Buddhist contexts, this argument is used to *rationally* reveal the *irrationality* of statements. It is applied to statements of various domains. However, for the context of the enactive approach, its application to part-whole relationships is especially interesting. As shown, autonomy and dynamic co-emergence exhibit a notion of relational co-origination between the parts and the whole (cf. section “The Enactive Approach and Its Reply to Foundationalism: Groundlessness”). The incoherence of this notion of relationality can be shown by applying the neither-one-nor-many argument as is done by John Dunne with reference to Dharmakīrti’s philosophy:^3^

*Dharmakīrti relies on a “neither-one-nor-many” argument to make his point, and his argument moves back and forth across a central question: if a relation is a real thing, then is it one with iths relata, or is it different from them? […] Dharmakīrti’s point is that, if a relation is different from the relata, then it must still somehow be distributed over them in order to serve its function as a relation. Hence, one may ask whether, by virtue of being distributed over the relata, the relation is thereby one with the relata, or different from them. If it is one, then there can be no relation, since relations presuppose multiplicity or plurality. And if it is different from the relata, then we must argue that there is some second-order relation that connects the relation to its relata. We can thus again ask: is this second-order relation one with its relata or different from them? The infinite regress from this point should be obvious (Dunne, 2004, pp. 43, 44)*.

Let us have a look on that argument step by step. As mentioned, the main assumption in relational approaches is that there are two or more things and a relation that ties them together. In case of co-emergence there are, for example, parts, the whole, and a relation between them (local-to-global determination and global-to-local determination). The key question of the neither-one-nor-many argument is whether the relation is the same as or different from the things it relates to each other (e.g., parts and whole).

Let us start with the assumption that they are the *same*. Is the relation the same as the things it ties together, i.e., is it connected to them? If it is the same, then the relation collapses into its relata. Then there is no relation anymore as it is the same as the things it is supposed to connect (Dunne, 2004, pp. 43, 44).

Therefore, if it is not the same, it is plausible to assume that the relation is different from the relata. Is the relation different from the things it ties together, i.e., is it unconnected to them? If it is different, the relation and the relata are *not* connected. Hence, there is a gap between the relation and the things it is supposed to connect. Accordingly, we need a relation that relates the relation to the relata. Otherwise, we get the gap again. Thereby, one needs to keep adding relations. This results in an infinite regress of relations. This is an inevitable problem with relationality.

In conclusion, the neither-one-nor-many argument deconstructs the idea of relationality. In this context, relationality appears to be incoherent. Relations mean that the relata and their relation in between would need to be the same (connected) and yet different (unconnected). From this perspective, relationality is paradoxical (Bitbol, 2005; cf. Dunne, 2004, pp. 43, 44).

Importantly, this argument does *not* prove relationality to be ultimately wrong. Rather, it illustrates a rational context in which this otherwise valid notion of relationality appears to be i*nvalid*. Therefore, statements are never ultimately true but context-dependent and observer-relative. Again, this applies not only to the enactive approach but even to this sentence itself!

For the context of this article, it is particularly interesting to emphasize this context-dependency and observer-relativity of the first level of analysis. The description of the first level of analysis (cf. section “First Level of Analysis”) cannot be ultimately true. All statements that I made concerning knowing groundlessness (RQ1) and concerning the transition process leading to it (RQ2) are necessarily context-dependent and observer-relative. They make only sense from a certain perspective. Furthermore, all statements in the first level of analysis are *arisen from within* a certain perspective. From which perspective has this first level of analysis arisen?

#### The Enacted Description Arises From Within Adaptive Sense-Making

As every statement is context-dependent and observer-relative, we may ask now about the context and the observer of the statements in this article: in which context and relative to which observer has the description of the first level of analysis arisen?

The description of the transition process from sense-making to knowing groundlessness is itself *from the perspective of sense-making*. It is clearly not from the perspective of knowing groundlessness which it tries to describe. This description is necessarily a description *of something*. Thereby, it is dual with a sense of a separation between the observer and the observed.

Remember the importance of recognizing the perspective from which this description has arisen. The aim of this description is to capture two different modes of knowing, namely sense-making and knowing groundlessness, and the transition between the two. However, the description itself is only from within *one* of the two perspectives. It has arisen *from within* sense-making.

To recognize this relativity of the description is highly insightful. It reveals this description’s bias. Through this reflexive act, we can now recognize the enacted description itself *as* an *act of enaction*. This makes one refraining from the sense that this description captures a perspective-independent reality. It does *not*. And that is alright. Finally, this opens the horizon and enables a shift of our perspective: what is knowing groundlessness *from within* knowing groundlessness?

#### The Enacted Description From the Perspective of Knowing Groundlessness

For gaining insight into knowing groundlessness, a description thereof from within knowing groundlessness seems important: what is knowing groundlessness *from within* knowing groundlessness? What is the transition process from sense-making to knowing groundlessness *from within* the “perspective” of knowing groundlessness?

The answer is simple but complicated: there is no meaningful answer to give. Why is this? Language is a certain tool. Necessarily, it comes with capabilities and constraints. One constraint is that descriptions involve the sense that they are descriptions *of something*. Thereby, language is intentional. It implies a subject-object duality. This ties language to sense-making (phase 1 enaction).

However, knowing groundlessness is *non*-intentional. It lacks any subject-object-duality. This unties it from sense-making (phase 1 enaction). Thereby, from within knowing groundlessness there is no object to describe and no subject that describes. Knowing groundlessness is mere unobscured non-dual knowing.

Therefore, in the context of the “perspective” from within knowing groundlessness, language is an inappropriate method. Any use of language reintroduces sense-making. This necessarily interrupts a moment of knowing groundlessness. Therefore, language and knowing groundlessness are mutually exclusive.

At this point, we have reached an end of linguistic description. Here, philosophical analysis is not useful anymore. From *within* this “perspective” of knowing groundlessness this exact knowing groundlessness can only be explored further when conceptual thinking is left behind.

#### Both Perspectives Are Context-Dependently Useful

The aim of this article is to describe the transition from sense-making to knowing groundlessness. The first level of analysis provided a description from *within* the perspective of sense-making. The second level of analysis provided the insight that this description is invalid from *within* the perspective of knowing groundlessness. Those two perspectives contradict each other.

However, this is not problematic at all. The contradiction simply exemplifies that there is not “one single” true view of reality. Rather, every view is context-dependent and observer-relative. Accordingly, the two views from the first and the second level of analysis can co-exist. They are *meaningful* or *true* in different contexts for different observers.

### Summary of the First and Second Level of Analysis

In this section we explored knowing groundlessness (RQ1) and the transition process leading to it (RQ2). We did so from two different perspectives: a first and a second level of analysis.

The first level of analysis provided a description from *within* the perspective of sense-making. In tackling the first research question, this first level of analysis resulted in a definition of knowing groundlessness: knowing groundlessness is phase 2 enaction knowing itself, unobscured by phase 1 enaction (adaptive sense-making). Thereby, knowing groundlessness is based on phase 2 enaction. Phase 2 enaction is an alternative mode of knowing that is non-intentional, self-known, and non-discerning. In a gap of phase 1 enaction, this phase 2 enaction is unobscured. In such a moment it can recognize itself. This unobscured self-recognition of phase 2 enaction is knowing groundlessness.

In tackling the second research question, the first level of analysis resulted in a description of this transition process. It exhibits two requirements: non-adaptivity and non-dual reflexive knowing (phase 2 enaction). Non-adaptivity is the first requirement: the living organism refrains from regulating itself with respect to the boundaries of its own viability. It does not appraise its experiences as good, bad, or irrelevant. Also, it does not approach, avoid, or ignore anything. Non-dual reflexive knowing (phase 2 enaction) is the second requirement. Via this phase 2 enaction, the living organism recognizes its current *acts* of sense-making and adaptivity. Those two requirements are met in the transition process from adaptive sense-making to knowing groundlessness. In this transition process the current acts of adaptivity and sense-making are experienced. Simultaneously, there is mere acceptance through which no adaptivity is added. Therefore, sense-making is decreased. Accordingly, when this is continued, acts of adaptivity and sense-making progressively decrease. Thereby, there is progressively less obscuration of the already present non-dual reflexive experiencing (phase 2 enaction). This may lead to a gap of sense-making in which phase 2 enaction can recognize itself. Experience experiences itself, non-dually. This is a moment of knowing groundlessness.

The second level of analysis provided the insight that this first level description is invalid from within the perspective of knowing groundlessness. The description is revealed as enacted. First, the provided description is context-dependent and observer-relative. Second, it comes from within acts of adaptive sense-making (phase 1 enaction). Third, this description is inappropriate from within the “perspective” of knowing groundlessness. Fourth, none of the two perspectives is ultimately true. However, they are valuable or *true* in different contexts.

## Discussion: A Vision for Cognitive Science

This article’s theoretical inquiry arises from within a certain epistemological framework. While this epistemological framework might substantially differ from the epistemological assumptions of many other scientific projects, it is crucially important to emphasize its *pluralistic* orientation. Instead of substituting the common epistemological grounding of cognitive science simply by just another doctrine, I rather wish to advocate the coexistence of various approaches and views. A more diverse landscape of different epistemological perspectives may valuably inform scientific inquiry by inspiring a variety of research questions, methodologies, and narratives in cognitive science. This article shall represent one of many expressions of this vision for a diverse landscape of perspectives.

From within the epistemological perspective of the enactive approach, one might argue that the quality of a scientific or philosophical article cannot be measured in terms of its degree to which it *represents* a context-*independent* and observer-*independent* reality. Rather, its quality appears to be tied to the meaning it has in certain contexts for certain communities of observers. Consequently, this perspective can be applied to the evaluation of this article’s quality. Therefore, the aim here is to evaluate in which contexts and for which observers those synthesized statements from the first and the second level of analysis might be meaningful or even valuable.

One specific context and community of observers is selected in this regard: *Cognitive scientists* who are willing to diversify and innovate cognitive science by providing alternative paradigms and perspectives. Accordingly, the more specific aim is to evaluate how the first and the second level of analysis can be meaningful in this context and for this community of observers in the cognitive sciences.

The respective question in this context is the following: How can the insights from the first and second level of analysis support a next generation of *cognitive scientists* who are willing to diversify cognitive science? What does this article hint at for a meaningful and responsible parallel paradigm in cognitive science?

Accordingly, I derive from this article some pointers to a vision of a meaningful paradigm in cognitive science: what could an additional cognitive science paradigm for illuminating an authentic understanding of the mind look like? At this point, I will allow myself to humbly envision some recommendations for one of many promising paradigmatic developments of cognitive science. However, it is important to emphasize that the aim is not to replace other approaches in cognitive science but to expand its perspectival repertoire. Therefore, the recommendations here are to be seen as pointing out *one* strand of development within a proposed *diverse* meshwork of views and methods within cognitive science.

First and foremost, what can be one overall aim of a genuine cognitive science? From my enaction-inspired perspective, this envisioned future of cognitive science may not aim primarily for technological artifacts or models of neural networks. Rather, it aims more at a (1) most *fundamental understanding* of the mind (2) in *direct experience*. First, a *fundamental understanding* of the mind implies that it is *really* about the *mind* and *consciousness* and not about something else which is confused with them (e.g., brains, computer programs, psychological models, and philosophical theories). This point concerns the *subject-matter* of this alternative paradigm in cognitive science. This article humbly contributed to that direction in tackling the first research question (RQ1): *what* is knowing groundlessness? Second, the *direct experience* of this subject-matter of *mind* and *consciousness* implies that it is *empirical*. Instead of theorizing about an abstracted notion of what *mind* and *consciousness* are (which this article necessarily is guilty of), they are directly *experienced*. This point concerns the *method* of this alternative paradigm in cognitive science. This article humbly contributed to that direction in tackling the second research question (RQ2): *how* does one shift from sense-making to knowing groundlessness?

In this regard, the here envisioned additional paradigm of cognitive science builds on the strong emphasis on the importance of directly experiencing what we are interested in: mind and consciousness. Accordingly, it is in close alignment with the previously cited quote by Evan Thompson which beautifully summarizes this point:

*It’s one thing to have a scientific representation of the mind as “enactive” – as embodied, emergent, dynamic, and relational; as not homuncular and skull-bound; and thus in a certain sense insubstantial. But it’s another thing to have a corresponding direct experience of this nature of the mind in one’s own first-person case (Thompson, 2004, p. 382)*.

How could such a radically different vision of cognitive science be implemented? How can we facilitate those required *direct* and *unobscured experiences* of *consciousness*? My current vision of an *authentic science of mind and consciousness* builds on two methodological pillars that might look radically different from classical cognitive science: (1) practicing to directly experience non-dual awareness and (2) communicating those experiences. First, it is required to investigate experiences of the *non-dual* from *within* experience. This means that such a vision for cognitive science includes the *embodied transformation* of the scientists who practice realizing the non-dual in their direct experience. This is a high aim as this kind of non-dual meditation practice requires extensive training. However, it may not require more training than what is required for enabling research in artificial intelligence or neuroscience. Therefore, the requirement for training in meditation practices is considered a minor hurdle.

Second, those experienced realizations of the non-dual need to be made *intersubjectively accessible*. In a current conception this can take two forms. On the one hand, those experiences are communicated as *direct descriptions of the non-dual*. On the other hand, *instructions* on *how* to realize the non-dual are communicated. This enables others to “empirically” test those experience descriptions within their own experience. In short, those experiences are communicated in the form of direct descriptions and of practice instructions.

In summary, while being embedded in a larger perspectival repertoire, my current vision of an authentic cognitive science requires the direct involvement of the cognitive scientist’s experience. The scientist becomes a practitioner: someone who trains to experience unobstructed experience itself and shares this intersubjectively with other members of his or her community. The aim is to build an additional cognitive science approach that is, first, *indeed* about the mind and consciousness and, second, *empirical* to the extent that it involves the direct unobstructed experience of the “object” of interest: mind and consciousness. Furthermore, since this “object” of interest is *not* an observed “object” but the observing mind itself, this authentic alternative cognitive science approach is to be envisioned as a science that goes *beyond* subject-object duality. It cannot be satisfied anymore with tackling something other than the intimate and immediate *experiencing* that gives rise to any knowledge. Therefore, this vision of an additional cognitive science paradigm consists in a *non-dual turn*. Instead of an abstract notion of a mind, the interest is concerning the mind in which the current experiences are happening “here right now”. Instead of pointing toward an abstract mind out there it investigates that which points. Thereby, this approach to cognitive science investigates the mind without making it an object. It investigates in a non-dual manner. And yet it shares those observations with others via experience descriptions and via practice instructions. To the best of my knowledge, this is what I envision as a genuine progress toward a vital and diverse science of consciousnesses and the mind.

## Data Availability Statement

The original contributions presented in the study are included in the article, further inquiries can be directed to the corresponding author.

## Author Contributions

The author confirms being the sole contributor of this work and has approved it for publication.

## Conflict of Interest

The author declares that the research was conducted in the absence of any commercial or financial relationships that could be construed as a potential conflict of interest.

## Publisher’s Note

All claims expressed in this article are solely those of the authors and do not necessarily represent those of their affiliated organizations, or those of the publisher, the editors and the reviewers. Any product that may be evaluated in this article, or claim that may be made by its manufacturer, is not guaranteed or endorsed by the publisher.

## Footnotes

1 This statement that reflexive non-dual knowing recognizes itself needs to be treated with caution. The way it is articulated may imply that it is a knowing *of* something, *of itself.* However, it is non-dual and therefore not separable in subject and object. Accordingly, in this mode of knowing there is no object to recognize. This reflexive non-dual knowing simply illuminates itself without any subject-object distinction.

2 From an enactive point of view, the context-dependency and observer-relativity of statements applies to information in general: “[f]or enactive theorists, information is context-dependent and agent-relative; it belongs to the coupling of a system and its environment” (Thompson, 2007, p. 51).

3 Although the following section is specifically dedicated to draw comparisons between the synthesis of this thesis and Buddhist philosophies, this preliminary reference to an ancient Buddhist argument is already here useful for evaluating the coherence of the notion of relationality.
